# Sunshine, temperature and suicidal behaviour in patients treated with antidepressants: an explorative nested case–control study

**DOI:** 10.1038/s41598-021-89499-4

**Published:** 2021-05-13

**Authors:** Georgios D. Makris, Richard A. White, Johan Reutfors, Lisa Ekselius, Morten Andersen, Fotios C. Papadopoulos

**Affiliations:** 1 Department of Neuroscience, Psychiatry, Uppsala University Hospital, Uppsala University, Uppsala, Sweden; 2 Norwegian Institute of Public Health, Oslo, Norway; 3 Centre for Pharmacoepidemiology, Department of Medicine, Karolinska University Hospital, Karolinska Institutet, Solna, Stockholm, Sweden; 4 Section of Pharmacotherapy, Department of Drug Design and Pharmacology, University of Copenhagen, Copenhagen, Denmark

**Keywords:** Risk factors, Depression, Epidemiology

## Abstract

Our aim was to explore if different exposure windows for sunshine or temperature are associated with increased suicidal behaviour among people starting antidepressant treatment. 307 completed and 1674 attempted suicides were included as cases in the conditional logistic regression analyses, while controlling for potential confounders, including season, as well as temperature and hours of sunshine when these variables were not the main exposure variable. Ten controls were matched to each case using risk-set sampling. The role of season, age, and sex was examined with likelihood ratio tests (LRTs) with and without the respective interaction terms and with stratified analyses. There was no overall association between temperature or sunshine with suicidal behaviour. Age was a significant effect modifier for suicide and suicide attempt for both sunshine and temperature exposure. In stratified analyses, an increase of one degree Celsius in the average daily temperature during the last 4 weeks was associated, in the unadjusted model, with a 3% increase in the rate of suicide (p = 0.023) amongst older patients (65+). In the same age group, an increase of 1 h in the average daily sunshine during the last 4 weeks was associated with an 8% increase in the rate of suicide attempt (p = 0.002), while the respective increase for the exposure period of 5–8 weeks was 7% (p = 0.007). An increase of one degree Celsius in the average daily temperature during the last 4 weeks was associated with a 3% increase in the rate of suicide attempt (p = 0.007). These associations did not retain statistical significance in the adjusted models. No associations were found in the other age groups. Our results point to a possible effect modification by age, with higher risk of suicidal behavior associated with an increase in sunshine and temperature found in the older age groups.

## Introduction

Demographers in the nineteenth century discovered that suicidal behavior peaks during the late spring and early summer period and declines during the mid-winter period^1^. On the other hand, the explanation of this phenomenon was a matter of debate. Two schools of thought evolved. The first one suggested that bioclimatic factors were of considerable importance^2^ and the second one attributed the seasonal pattern to socioeconomic factors^3^.

First it was suggested that light could possibly interfere with the endocrine system thereby affecting processes towards suicidal behaviour^4^. Later, other researchers suggested that the seasonal incidence of suicide is affected by temperature^5^. Many studies thereafter have examined the possible association of weather parameters and suicide seasonality. The majority of the studies have found an association but the results have been contradictory^6,7^. The two most widely studied variables are temperature and sunlight, but the underlying mechanism for how these climatic variables may trigger suicidal behavior is far from clear.

Serotonergic neurotransmission has been suggested to be central in the phenomenon of suicide seasonality, mainly due to the fact that this system is central in the pathophysiology of suicide and has also been found to follow a circannual rhythm^8–12^. Changes of several serotonin-related measures in plasma and whole blood of healthy individuals have been reported to vary throughout the year, with maximum values during the summer and lowest values in the fall^8,9^. However, 5-hydroxyindoleacetic acid (5-HIAA) in CSF follows an opposite seasonal pattern. Serotonin turnover assessed by measuring concentrations of serotonin in the internal jugular vein in healthy individuals was found to be lowest in the winter and inversely associated with duration of bright sunlight^9^. Moreover, some positron emission tomography and single-photon emission computed tomography studies in healthy and depressed individuals have reported that the serotonin transporter (SERT) presents an increased binding capacity in winter and decreased capacity in the spring^10,11^, although other researchers could not verify this finding^12^. Several lines of evidence from post-mortem studies of suicide victims, as well as in vivo imaging studies of suicide attempters suggest that serotonergic neurotransmission is implicated in the suicidal process, possibly through a decreased SERT binding in the prefrontal cortex^13^. The reported seasonal variation of SERT binding capacity coincides with the seasonal peak of suicide in the spring and early summer that has been reported in many ecological studies from different countries^14^.

Antidepressants appear to exert their pharmacological action by modulating serotonergic neurotransmission and their efficacy for the treatment of depressive and anxiety disorders has been demonstrated, but also the potential adverse effect of triggering suicidal behavior in younger people^15^. We have previously reported an increased seasonal pattern in suicide victims with positive forensic screening for antidepressants in blood at the time of suicide^16^, as well as a stronger association between sunshine and suicide among both men and women with positive forensic screening for selective serotonin reuptake inhibitors (SSRIs), even after adjustment for season and time trend for suicide, compared to those not on SSRIs^17^.

A population on antidepressants may thus be advantageous in studying associations between climatic variables and suicidal behaviour. Our aim was to explore if different exposure windows for sunshine or temperature are associated with increased suicidal behaviour, measured by suicide and attempted suicide, among people starting an antidepressant treatment. Our hypothesis was that exposure to higher sunshine or temperature would be associated with an increased risk of suicidal behavior.

## Results

We identified individuals that redeemed a prescription of at least one antidepressant between July 2006 and December 2012. Patients who had redeemed another prescription (antidepressants, antipsychotics, mood stabilizers), had been hospitalized the previous year, or had invalid information in the prescription register were excluded. Those that were lacking information about county of residence were also excluded. Data about temperature and sunshine duration were obtained from the Swedish Meteorological and Hydrological Institute. We used a nested case control design and conditional logistic regression analyses. Completed and attempted suicides were included as cases. Ten controls were matched to each case using risk-set sampling. The mean daily temperature and the mean daily sunshine over the weeks 1–4 and 5–8 before suicide or suicide attempt were the main exposure variables. We used three models in our analyses: (1) crude model with no confounders and a single exposure (sunshine or temperature), (2) base model with confounders such as history of previous suicide-attempt, county, year and season and (3) climatic model, which is the base model with an additional exposure of the other climatic variable. The role of season, age, and sex was examined with likelihood ratio tests (LRTs) with and without the respective interaction terms and with stratified analyses.

There were 307 suicides and 1674 suicide attempts during the study period. Table 1 presents the distribution of age, sex, prescription setting, previous suicide attempt and type of antidepressant medication in cases (suicides and suicide-attempts) and controls.

**Table 1:** The distribution of age, sex, prescription setting, previous suicide attempt and type of antidepressant medication in cases (suicides and suicide-attempts) and controls within different strata. For each case, 10 controls were randomly sampled from the population at risk.

	N		Age (mean, sd)		Female %		Prescription in primary care (%)		Previous suicide attempts (%)		Prescribed SSRIs (%)	
	Cases	Controls	Cases	Controls	Cases	Controls	Cases	Controls	Cases	Controls	Cases	Controls
**Suicides**	307	3070	56.1 (18.9)	51.2 (20.9)	23.5	62.9	65.5	69.8	5.2	1.2	69.1	67.0
Male	235	2350	56.9 (18.4)	52.1 (20.5)	0.0	0.0	63.8	67.1	3.8	0.9	69.4	63.8
Female	21	210	20.5 (2.6)	19.8 (3.5)	28.6	64.3	38.1	46.2	4.8	1.9	66.7	77.6
Age < 25	177	1770	48.3 (11.4)	45.0 (11.1)	23.7	62.1	65.0	71.4	6.2	1.6	69.5	64.9
Age 25–64	109	1090	75.6 (8.4)	77.7 (8.6)	22.0	63.9	71.6	73.9	3.7	0.3	68.8	63.7
Age 65 +	15	150	20.7 (2.4)	19.2 (3.7)	0.0	0.0	33.3	42.3	6.7	1.3	73.3	76.7
*Treatment start*												
Winter	135	1350	49.5 (11.5)	44.9 (11.2)	0.0	0.0	63.7	69.3	4.4	1.2	68.1	64.6
Spring	85	850	75.1 (8.4)	77.2 (7.7)	0.0	0.0	69.4	70.8	2.4	0.5	70.6	63.3
Summer	42	420	44.5 (10.5)	43.9 (11.1)	100.0	100.0	69.0	70.0	11.9	1.7	73.8	69.5
Autumn	24	240	77.3 (8.3)	77.4 (8.4)	100.0	100.0	79.2	75.4	8.3	1.2	62.5	64.6
**Suicide attempts**	42	420	50.8 (17.9)	49.6 (20.8)	47.6	64.5	52.4	66.8	11.9	1.9	66.7	70.2
Male	22	220	49.1 (16.3)	52.1 (21.2)	0.0	0.0	45.5	64.5	13.6	1.4	68.2	61.8
Female	20	200	52.7 (19.9)	53.3 (20.6)	100.0	100.0	60.0	64.3	10.0	1.5	65.0	71.0
Age < 25	82	820	55.2 (19.2)	51.4 (21.3)	30.5	64.6	67.1	69.1	2.4	1.0	64.6	65.1
Age 25–64	86	860	54.4 (19.3)	50.5 (19.8)	19.8	63.0	69.8	70.5	4.7	1.5	75.6	68.7
Age 65 +	75	750	59.4 (18.0)	52.0 (20.8)	21.3	61.2	64.0	68.0	6.7	0.7	72.0	68.3
*Treatment start*												
Winter	64	640	55.6 (19.0)	49.8 (20.6)	21.9	65.6	59.4	69.2	7.8	1.1	62.5	67.3
Spring	1674	16,740	33.8 (18.9)	51.4 (20.6)	64.6	63.4	44.4	69.8	13.6	1.5	76.4	66.3
Summer	593	5930	38.4 (20.1)	51.1 (20.5)	0.0	0.0	47.5	67.0	10.8	1.3	68.3	64.6
Autumn	1081	10,810	31.2 (17.7)	51.4 (20.8)	100.0	100.0	42.7	71.6	15.2	1.7	80.9	67.4

**Figure 1:**
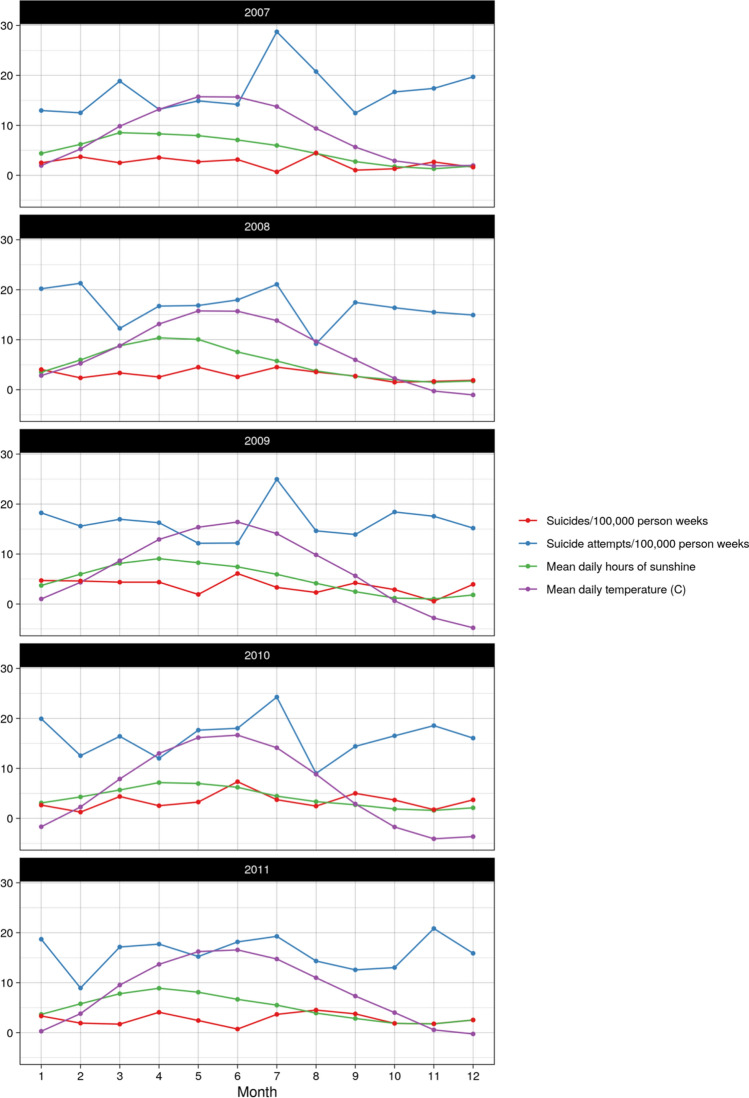
Rates of suicide and attempted suicide, as well as the mean daily temperature and sunshine hours by year, during the study period in our source population.

Figure 1 presents the rates of suicide and attempted suicide, as well as the mean daily temperature and sunshine hours during the study period in our source population.

### Suicides

We found no evidence of an association between the outcome of suicide and the exposures of sunshine and temperature in the main analyses in all three models (Table 2). There was evidence of effect modification by age in suicides (Table 3), with the LRTs for the interaction term of temperature reaching statistical significance (p = 0.003) for the daily average temperature during the weeks 1–4 before the index and for the window of 5–8 weeks (p = 0.005). Similarly, evidence of effect modification by age was found for the exposure of sunshine during the window 5–8 weeks (p = 0.04). No evidence of effect modification was found for either season or sex in suicide for the exposure of sunshine or temperature in both exposure windows (1–4 and 5–8 weeks before the index).

**Table 2:** Conditional logistic regression derived odds ratios (ORs) and 95% confidence intervals (95% CIs) for suicide and suicide attempt associated with sunshine hours and temperature during two exposure windows.

Exposure and exposure time window	Exposure (mean, SD)		OR (95% CI)		
	Cases	Controls	Crude	Base**	Climate adjusted***
	Suicide				
	(N = 307)	(N = 3070)			
**Sunshine (mean hours)**					
1–4 weeks	4.96 (3.26)	4.80 (3.24)	1.02 (0.98–1.05); p = 0.408	1.01 (0.96–1.07); p = 0.670	1.01 (0.95–1.08); p = 0.708
5–8 weeks	4.66 (3.11)	4.75 (3.28)	0.99 (0.96–1.03); p = 0.637	0.98 (0.93–1.03); p = 0.413	0.99 (0.92–1.05); p = 0.675
**Temperature (mean °C)**					
1–4 weeks	7.24 (7.82)	7.11 (7.24)	1.00 (0.99–1.02); p = 0.750	1.00 (0.98–1.03); p = 0.813	1.00 (0.97–1.03); p = 0.990
5–8 weeks	6.61 (7.69)	7.13 (7.40)	0.99 (0.98–1.01); p = 0.243	0.99 (0.96–1.01); p = 0.362	0.99 (0.96–1.02); p = 0.603

Exposure and exposure time window	Exposure (mean, SD)		OR (95% CI)		
	Cases	Controls	Crude	Base**	Climate adjusted***
	Suicide attempt				
	(N = 1674)	(N = 16,740)			
**Sunshine (mean hours)**					
1–4 weeks	4.72 (3.29)	4.81 (3.29)	0.99 (0.98–1.01); p = 0.303	0.99 (0.97–1.01); p = 0.458	0.99 (0.97–1.02); p = 0.728
5–8 weeks	4.70 (3.23)	4.72 (3.21)	1.00 (0.98–1.01); p = 0.765	1.00 (0.98–1.02); p = 0.998	1.02 (0.99–1.05); p = 0.234
**Temperature (mean °C)**					
1–4 weeks	6.91 (7.46)	7.01 (7.37)	1.00 (0.99–1.00); p = 0.523	1.00 (0.98–1.01); p = 0.423	1.00 (0.98–1.01); p = 0.665
5–8 weeks	6.86 (7.35)	7.00 (7.30)	1.00 (0.99–1.00); p = 0.327	0.99 (0.98–1.00); p = 0.113	0.99 (0.97–1.00); **p = 0.048**

Statistically significant result is given in bold.

**Adjusted for previous suicide attempt, county, sex, age, year and season when the antidepressant treatment was initiated.

***Adjusted for all variables in Base model and temperature when sunshine was the exposure variable or sunshine when temperature was the exposure variable.

**Table 3:** P-values for likelihood ratio tests (LRTs) testing the models including interaction terms (e.g. season of treatment initiation × average daily sunshine 1–4 weeks) against the models without the interaction terms. All models were adjusted for previous suicide-attempt, county, sex, age, year and season when the antidepressant treatment was initiated.

LRTs derived p-values for the interaction terms					
	Interaction term	Sunshine 1–4 weeks	Sunshine 5–8 weeks	Temperature 1–4 weeks	Temperature 5–8 weeks
Suicides	Season	0.778	0.501	0.445	0.298
	Age	0.117	**0.041**	**0.003**	**0.005**
	Sex	0.814	0.633	0.256	0.712
Suicide attempts	Season	0.073	0.482	0.411	0.790
	Age	**0.003**	**0.044**	**0.013**	0.143
	Sex	0.282	0.397	0.889	0.608

P-values < 0.05 are given in bold.

In stratified analyses (Table 4), no association between suicide and the exposure of sunshine and temperature was found in either sex or the age groups 0–24 and 25–64.

**Table 4:** Conditional logistic regression odds ratios (ORs) and 95% confidence intervals (95% CIs) for suicide associated with sunshine hours and temperature during two exposure windows, by sex, age and season when the antidepressant treatment was started.

Exposure and exposure time window		Exposure (mean, SD)		OR (95% CI)		
		Cases (suicide)	Controls	Crude	Base**	Climate adjusted***
**Males**		**(N = 235)**	**(N = 2350)**			
Sunshine (mean hours)	1–4 weeks	4.98 (3.18)	4.63 (3.21)	1.03 (0.99–1.08); p = 0.119	1.02 (0.96–1.09); p = 0.529	1.02 (0.94–1.10); p = 0.669
	5–8 weeks	4.72 (3.11)	4.56 (3.12)	1.02 (0.97–1.06); p = 0.465	0.99 (0.93–1.05); p = 0.660	1.02 (0.94–1.09); p = 0.697
Temperature (mean °C)	1–4 weeks	7.42 (7.78)	6.68 (7.34)	1.01 (1.00–1.03); p = 0.129	1.01 (0.98–1.04); p = 0.578	1.00 (0.97–1.04); p = 0.849
	5–8 weeks	6.59 (7.78)	6.90 (7.31)	0.99 (0.98–1.01); p = 0.551	0.98 (0.95–1.01); p = 0.181	0.98 (0.94–1.01); p = 0.186
**Females**		**(N = 72)**	**(N = 720)**			
Sunshine (mean hours)	1–4 weeks	4.93 (3.55)	4.72 (3.30)	1.02 (0.95–1.09); p = 0.622	1.05 (0.94–1.17); p = 0.387	1.04 (0.91–1.19); p = 0.588
	5–8 weeks	4.47 (3.14)	4.64 (3.30)	0.98 (0.91–1.06); p = 0.677	0.98 (0.87–1.10); p = 0.682	0.93 (0.80–1.07); p = 0.315
Temperature (mean °C)	1–4 weeks	6.65 (7.99)	6.44 (7.49)	1.00 (0.97–1.04); p = 0.814	1.02 (0.96–1.08); p = 0.483	1.01 (0.94–1.08); p = 0.810
	5–8 weeks	6.69 (7.41)	6.53 (7.43)	1.00 (0.97–1.04); p = 0.780	1.02 (0.97–1.08); p = 0.456	1.04 (0.97–1.12); p = 0.238
**Age < 25**		**(N = 21)**	**(N = 210)**			
Sunshine (mean hours)	1–4 weeks	4.37 (3.29)	4.79 (3.31)	0.96 (0.83–1.11); p = 0.570	0.89 (0.72–1.11); p = 0.304	0.98 (0.72–1.34); p = 0.908
	5–8 weeks	4.09 (2.73)	4.49 (3.18)	0.96 (0.83–1.11); p = 0.568	0.89 (0.71–1.12); p = 0.326	1.02 (0.75–1.41); p = 0.879
Temperature (mean °C)	1–4 weeks	5.07 (9.12)	6.63 (7.19)	0.97 (0.91–1.03); p = 0.335	0.93 (0.85–1.02); p = 0.124	0.93 (0.82–1.07); p = 0.309
	5–8 weeks	4.32 (7.84)	6.50 (7.59)	0.96 (0.91–1.02); p = 0.208	0.91 (0.83–1.01); p = 0.077	0.91 (0.79–1.04); p = 0.162
**Age 25**–**64**		**(N = 177)**	**(N = 1770)**			
Sunshine (mean hours)	1–4 weeks	4.81 (3.23)	4.71 (3.28)	1.01 (0.96–1.06); p = 0.700	0.99 (0.92–1.06); p = 0.810	1.00 (0.92–1.09); p = 0.963
	5–8 weeks	4.42 (3.07)	4.59 (3.13)	0.98 (0.93–1.03); p = 0.499	0.98 (0.91–1.05); p = 0.524	0.98 (0.89–1.07); p = 0.600
Temperature (mean °C)	1–4 weeks	6.46 (7.83)	6.72 (7.33)	0.99 (0.97–1.02); p = 0.612	0.99 (0.96–1.03); p = 0.667	0.99 (0.95–1.04); p = 0.714
	5–8 weeks	5.98 (7.59)	6.84 (7.29)	0.98 (0.96–1.01); p = 0.142	0.99 (0.96–1.03); p = 0.692	1.00 (0.96–1.05); p = 0.993
**Age 65 + **		**(N = 109)**	**(N = 1090)**			
Sunshine (mean hours)	1–4 weeks	5.33 (3.30)	4.94 (3.24)	1.04 (0.98–1.10); p = 0.228	1.06 (0.97–1.16); p = 0.210	1.02 (0.91–1.13); p = 0.769
	5–8 weeks	5.15 (3.21)	4.73 (3.19)	1.04 (0.98–1.11); p = 0.177	1.04 (0.95–1.15); p = 0.400	1.03 (0.92–1.15); p = 0.588
Temperature (mean °C)	1–4 weeks	8.94 (7.28)	7.22 (7.38)	**1.03 (1.00–1.06); p = 0.023**	1.05 (1.00–1.10); p = 0.063	1.04 (0.98–1.11); p = 0.163
	5–8 weeks	8.07 (7.63)	6.95 (7.37)	1.02 (0.99–1.05); p = 0.142	1.02 (0.97–1.07); p = 0.470	1.01 (0.96–1.07); p = 0.741
**Winter**		**(N = 82)**	**(N = 820)**			
Sunshine (mean hours)	1–4 weeks	2.27 (2.22)	2.27 (1.69)	1.00 (0.85–1.17); p = 0.985	0.99 (0.84–1.17); p = 0.944	1.03 (0.86–1.23); p = 0.756
	5–8 weeks	1.71 (1.14)	1.68 (0.91)	1.04 (0.80–1.35); p = 0.781	1.01 (0.76–1.35); p = 0.926	1.02 (0.77–1.35); p = 0.900
Temperature (mean °C)	1–4 weeks	–0.88 (4.99)	–0.45 (4.27)	0.97 (0.92–1.03); p = 0.340	0.97 (0.91–1.05); p = 0.460	0.97 (0.90–1.05); p = 0.423
	5–8 weeks	–0.09 (4.18)	0.18 (4.48)	0.98 (0.94–1.04); p = 0.545	0.99 (0.92–1.06); p = 0.702	0.99 (0.92–1.06); p = 0.695
**Spring**		**(N = 86)**	**(N = 860)**			
Sunshine (mean hours)	1–4 weeks	7.19 (2.97)	7.00 (2.94)	1.03 (0.95–1.11); p = 0.540	1.05 (0.96–1.14); p = 0.279	1.06 (0.94–1.19); p = 0.345
	5–8 weeks	5.00 (2.87)	5.13 (3.03)	0.98 (0.90–1.07); p = 0.658	0.99 (0.91–1.09); p = 0.870	1.03 (0.90–1.18); p = 0.698
Temperature (mean °C)	1–4 weeks	7.58 (5.83)	7.59 (5.56)	1.00 (0.95–1.05); p = 0.953	1.02 (0.96–1.07); p = 0.570	0.99 (0.92–1.07); p = 0.815
	5–8 weeks	3.05 (5.89)	3.64 (5.59)	0.98 (0.93–1.02); p = 0.296	0.98 (0.93–1.04); p = 0.573	0.97 (0.90–1.06); p = 0.507
**Summer**		**(N = 75)**	**(N = 750)**			
Sunshine (mean hours)	1–4 weeks	7.02 (2.20)	7.33 (2.39)	0.94 (0.84–1.04); p = 0.237	0.96 (0.85–1.09); p = 0.540	0.94 (0.83–1.07); p = 0.346
	5–8 weeks	8.02 (2.22)	8.22 (2.36)	0.96 (0.86–1.07); p = 0.457	1.00 (0.88–1.13); p = 0.999	1.00 (0.88–1.14); p = 0.988
Temperature (mean °C)	1–4 weeks	15.87 (2.79)	15.68 (2.65)	1.03 (0.94–1.14); p = 0.488	1.05 (0.94–1.17); p = 0.376	1.07 (0.95–1.19); p = 0.257
	5–8 weeks	14.68 (3.53)	14.79 (3.14)	0.98 (0.91–1.07); p = 0.703	0.99 (0.91–1.07); p = 0.746	0.99 (0.90–1.08); p = 0.747
**Autumn**		**(N = 64)**	**(N = 640)**			
Sunshine (mean hours)	1–4 weeks	3.02 (1.64)	2.85 (1.67)	1.08 (0.91–1.30); p = 0.374	1.19 (0.97–1.45); p = 0.100	1.07 (0.83–1.38); p = 0.589
	5–8 weeks	4.04 (1.76)	3.98 (1.92)	1.02 (0.87–1.20); p = 0.773	1.16 (0.96–1.40); p = 0.121	1.05 (0.81–1.35); p = 0.721
Temperature (mean °C)	1–4 weeks	6.95 (5.98)	5.81 (5.41)	**1.08 (1.01–1.15); p = 0.027**	**1.08 (1.00–1.16); p = 0.044**	1.06 (0.97–1.16); p = 0.182
	5–8 weeks	10.65 (5.78)	9.69 (5.33)	**1.07 (1.00–1.15); p = 0.045**	1.07 (0.99–1.15); p = 0.074	1.06 (0.96–1.17); p = 0.267

Statistically significant results are given in bold.

**Adjusted for previous suicide attempt, county, sex, age, year and season when the antidepressant treatment was initiated.

***Adjusted for all variables in Base model and temperature when sunshine was the exposure variable or sunshine when temperature was the exposure variable.

The only positive association was found in the crude model between the average daily temperature during the last 4 weeks and completed suicide (crude OR = 1.03, 95% CI 1.00–1.06, p = 0.023) amongst older patients (65+). However, this association was no longer statistically significant when adjusting for confounders such as previous suicide-attempt, county, season and year (adjusted OR = 1.05, 95% CI 1.00–1.10, p = 0.06). In the stratified analyses by season, there was a positive association between temperature and suicide in autumn, with an 8% increased risk in suicide risk for each increase in daily average temperature with one degree during the weeks 1–4 before index (crude OR = 1.08, 95% CI 1.01–1.15, p = 0.027), which remained significant when adjusting for confounders in the base model but not when adjusting for sunshine (climate model). Similar pattern was seen for the exposure of temperature in autumn 5–8 weeks before the index.

### Suicide attempts

We found no evidence of an association between the outcome of suicide attempts and the exposures of sunshine and temperature in the main analyses in all three models (Table 2). There was statistically significant effect modification by age in suicide attempts for the exposures of daily average sunshine during the past 1–4 weeks (p = 0.003) and the period 5–8 weeks before the suicide attempt (p = 0.04), as well as for the exposure of daily average temperature during the past 1–4 weeks (p = 0.013) (Table 3). No evidence of effect modification by either season or sex in suicide attempts was found for the exposure of sunshine or temperature in both exposure windows (1–4 and 5–8 weeks before the index) (Table 3).

In stratified analyses by sex (Table 5), no consistent association between suicide attempt and the exposure of sunshine and temperature was found. In the stratified analyses by age, in the older age group (65+) an increase of 1 h in the average daily sunshine during the last 4 weeks was associated with an 8% increase in the risk of suicide attempt (crude OR = 1.08, 95% CI 1.03–1.13, p = 0.002). Similarly, an increase of 1 h in the average daily sunshine during the weeks 5–8 was associated with a 7% increased risk of suicide attempt (crude OR = 1.07, 95% CI 1.02–1.13, p = 0.007). In the same age group, an increase of one degree Celsius in the daily average temperature during the weeks 1–4 was associated with a 3% increase of suicide risk (crude OR = 1.03, 95% CI 1.01–1.06, p = 0.016) (Table 5). These associations were no longer statistically significant after adjusting in the base and climate model.

**Table 5:** Conditional logistic regression odds ratios (ORs) and 95% confidence intervals (95% CIs) for suicide attempt associated with sunshine hours and temperature during two exposure windows, by sex, age and season when the antidepressant treatment was started.

Exposure and exposure time window		Exposure (mean, SD)		OR (95% CI)		
		Cases (suicide attempt)	Controls	Crude	Base**	Climate adjusted***
**Males**		**(N = 593)**	**(N = 5930)**			
Sunshine (mean hours)	1–4 weeks	4.79 (3.33)	4.74 (3.27)	1.00 (0.98–1.03); p = 0.707	1.00 (0.96–1.04); p = 0.941	1.03 (0.98–1.08); p = 0.305
	5–8 weeks	4.78 (3.34)	4.70 (3.21)	1.01 (0.98–1.04); p = 0.534	1.00 (0.96–1.04); p = 0.938	1.04 (0.99–1.09); p = 0.118
Temperature (mean °C)	1–4 weeks	6.97 (7.55)	6.98 (7.35)	1.00 (0.99–1.01); p = 0.882	0.99 (0.97–1.01); p = 0.203	0.98 (0.96–1.00); p = 0.103
	5–8 weeks	6.77 (7.50)	6.99 (7.35)	1.00 (0.98–1.01); p = 0.429	**0.98 (0.96–1.00); p = 0.039**	**0.97 (0.95–0.99); p = 0.010**
**Females**		**(N = 1081)**	**(N = 10,810)**			
Sunshine (mean hours)	1–4 weeks	4.68 (3.27)	4.75 (3.31)	0.99 (0.98–1.01); p = 0.534	1.00 (0.97–1.03); p = 0.837	1.00 (0.97–1.04); p = 0.931
	5–8 weeks	4.65 (3.16)	4.71 (3.25)	0.99 (0.98–1.01); p = 0.606	1.00 (0.97–1.03); p = 0.841	1.00 (0.97–1.04); p = 0.836
Temperature (mean °C)	1–4 weeks	6.87 (7.42)	6.85 (7.37)	1.00 (0.99–1.01); p = 0.777	1.00 (0.99–1.02); p = 0.823	1.00 (0.98–1.02); p = 0.901
	5–8 weeks	6.90 (7.27)	6.91 (7.37)	1.00 (0.99–1.01); p = 0.801	1.00 (0.98–1.01); p = 0.490	0.99 (0.98–1.01); p = 0.501
**Age < 25**		**(N = 764)**	**(N = 7640)**			
Sunshine (mean hours)	1–4 weeks	4.40 (3.27)	4.60 (3.25)	0.98 (0.96–1.00); p = 0.106	1.00 (0.96–1.03); p = 0.909	1.00 (0.95–1.04); p = 0.921
	5–8 weeks	4.46 (3.15)	4.56 (3.16)	0.99 (0.97–1.01); p = 0.386	1.00 (0.97–1.04); p = 0.953	1.02 (0.97–1.06); p = 0.456
Temperature (mean °C)	1–4 weeks	6.28 (7.31)	6.46 (7.49)	1.00 (0.99–1.01); p = 0.468	1.00 (0.98–1.02); p = 0.932	1.00 (0.98–1.02); p = 0.993
	5–8 weeks	6.51 (7.32)	6.59 (7.40)	1.00 (0.99–1.01); p = 0.804	0.99 (0.98–1.01); p = 0.385	0.99 (0.97–1.01); p = 0.253
**Age 25**–**64**		**(N = 754)**	**(N = 7540)**			
Sunshine (mean hours)	1–4 weeks	4.85 (3.26)	4.69 (3.25)	1.02 (0.99–1.04); p = 0.189	1.01 (0.98–1.04); p = 0.544	1.02 (0.98–1.07); p = 0.353
	5–8 weeks	4.80 (3.24)	4.61 (3.20)	1.02 (1.00–1.04); p = 0.124	1.02 (0.99–1.06); p = 0.267	1.04 (0.99–1.08); p = 0.100
Temperature (mean °C)	1–4 weeks	7.18 (7.54)	6.84 (7.32)	1.01 (0.99–1.02); p = 0.311	1.00 (0.98–1.02); p = 0.916	0.99 (0.97–1.01); p = 0.509
	5–8 weeks	7.00 (7.45)	6.85 (7.37)	1.00 (0.99–1.01); p = 0.918	1.00 (0.98–1.01); p = 0.739	0.99 (0.97–1.01); p = 0.206
**Age 65 + **		**(N = 156)**	**(N = 1560)**			
Sunshine (mean hours)	1–4 weeks	5.70 (3.29)	4.82 (3.30)	**1.08 (1.03–1.13); p = 0.002**	1.04 (0.97–1.13); p = 0.273	1.05 (0.96–1.16); p = 0.266
	5–8 weeks	5.40 (3.44)	4.69 (3.14)	**1.07 (1.02–1.13); p = 0.007**	1.03 (0.95–1.11); p = 0.493	1.04 (0.95–1.14); p = 0.419
Temperature (mean °C)	1–4 weeks	8.70 (7.45)	7.06 (7.27)	**1.03 (1.01–1.06); p = 0.016**	1.01 (0.97–1.05); p = 0.760	0.99 (0.94–1.04); p = 0.684
	5–8 weeks	7.88 (6.95)	6.91 (7.26)	1.02 (0.99–1.04); p = 0.182	1.00 (0.96–1.04); p = 0.945	0.99 (0.94–1.04); p = 0.673
**Winter**		**(N = 437)**	**(N = 4370)**			
Sunshine (mean hours)	1–4 weeks	2.22 (1.77)	2.39 (1.96)	**0.93 (0.86–0.99); p = 0.024**	**0.93 (0.87–0.99); p = 0.034**	0.93 (0.87–1.01); p = 0.087
	5–8 weeks	1.66 (1.03)	1.76 (1.08)	0.90 (0.81–1.00); p = 0.050	0.90 (0.81–1.01); p = 0.074	0.91 (0.81–1.02); p = 0.099
Temperature (mean °C)	1–4 weeks	–0.35 (4.51)	–0.30 (4.44)	0.99 (0.97–1.02); p = 0.584	0.98 (0.95–1.01); p = 0.181	0.99 (0.96–1.03); p = 0.669
	5–8 weeks	0.07 (4.42)	0.04 (4.45)	1.00 (0.97–1.02); p = 0.851	0.99 (0.96–1.02); p = 0.354	0.99 (0.96–1.02); p = 0.541
**Spring**		**(N = 422)**	**(N = 4220)**			
Sunshine (mean hours)	1–4 weeks	7.47 (2.85)	7.46 (2.86)	1.00 (0.96–1.04); p = 0.940	1.01 (0.97–1.06); p = 0.486	1.03 (0.97–1.08); p = 0.335
	5–8 weeks	5.92 (3.15)	6.00 (3.25)	0.99 (0.95–1.03); p = 0.524	1.00 (0.96–1.04); p = 0.893	1.05 (0.99–1.11); p = 0.131
Temperature (mean °C)	1–4 weeks	8.94 (5.72)	9.06 (5.77)	1.00 (0.97–1.02); p = 0.781	1.00 (0.97–1.02); p = 0.919	0.99 (0.96–1.02); p = 0.490
	5–8 weeks	4.76 (6.11)	5.23 (6.09)	0.98 (0.96–1.00); p = 0.107	0.98 (0.96–1.01); p = 0.127	**0.96 (0.93–1.00); p = 0.032**
**Summer**		**(N = 372)**	**(N = 3720)**			
Sunshine (mean hours)	1–4 weeks	7.05 (2.44)	6.90 (2.49)	1.03 (0.98–1.09); p = 0.184	1.05 (0.99–1.10); p = 0.116	1.03 (0.97–1.09); p = 0.377
	5–8 weeks	8.02 (2.32)	7.93 (2.45)	1.02 (0.97–1.07); p = 0.480	1.03 (0.98–1.08); p = 0.293	1.03 (0.97–1.08); p = 0.340
Temperature (mean °C)	1–4 weeks	15.53 (3.05)	15.20 (3.24)	1.03 (0.99–1.08); p = 0.098	1.04 (0.99–1.08); p = 0.093	1.03 (0.98–1.08); p = 0.226
	5–8 weeks	15.18 (2.84)	15.07 (3.05)	1.01 (0.97–1.05); p = 0.607	1.01 (0.97–1.05); p = 0.740	1.00 (0.96–1.05); p = 0.895
**Autumn**		**(N = 443)**	**(N = 4430)**			
Sunshine (mean hours)	1–4 weeks	2.62 (1.63)	2.62 (1.63)	1.00 (0.93–1.07); p = 0.958	0.99 (0.92–1.07); p = 0.893	1.06 (0.96–1.16); p = 0.270
	5–8 weeks	3.75 (1.92)	3.76 (1.94)	0.99 (0.93–1.06); p = 0.839	1.00 (0.93–1.07); p = 0.986	1.08 (0.98–1.18); p = 0.130
Temperature (mean °C)	1–4 weeks	4.86 (5.36)	5.18 (5.44)	0.98 (0.96–1.01); p = 0.170	0.98 (0.96–1.01); p = 0.133	0.97 (0.94–1.00); p = 0.061
	5–8 weeks	8.55 (5.62)	8.92 (5.53)	0.98 (0.96–1.01); p = 0.128	0.98 (0.95–1.00); p = 0.086	**0.96 (0.93–0.99); p = 0.021**

Statistically significant results are given in bold.

**Adjusted for previous suicide attempt, county, sex, age, year and season when the antidepressant treatment was initiated.

***Adjusted for all variables in Base model and temperature when sunshine was the exposure variable or sunshine when temperature was the exposure variable.

In the age groups of 0–24 and 25–64 no association between suicide attempt and the exposure of sunshine and temperature was found. In the stratified analyses by season, there was a negative association between sunshine and suicide attempts in winter, with a 7% decreased risk in the of suicide attempt for each increase in daily average sunshine with 1 h (crude OR = 0.93, 95% CI 0.86–0.99, p = 0.024), which remained significant when adjusting for confounders in the base model but not when adjusting for temperature (climate model).

## Discussion

In this population of patients starting on antidepressant medication, no robust overall associations were found between sunshine and temperature and suicidal behaviour. However, there was evidence of effect modification of age mainly in attempted suicides with a similar trend in completed suicides. Our results suggest a positive (harmful) association between average daily temperature during the last 4 weeks and the risk of suicide attempt among older patients, while the harmful association between average daily sunshine and suicide attempt in the same age group was evident during both the 4 weeks prior but even during the weeks 5–8 before the event.

Temperature has been positively associated with suicide in a number of studies, while negatively in others^6,7,18,19^. Some recent systematic review and meta-analyses have found strong association between higher ambient temperature and suicide^7,19,20^. Sunlight has also been reported to have a positive association with suicide in the majority of studies^21^, while some studies have reported a negative association^22^, and others have reported no association at all^23^. A plausible reason for the inconsistency between studies may be differences in methodology. Apart from the fact that different studies deal with different number and type of weather variables, there is not a unified hypothesis on whether climate has an acute or more chronic effect on suicide and consequently daily, weekly, monthly or annual meteorological data have been used. Most important though is that several studies that find associations do not adjust for the effect of season or other climate factors, thus making impossible to disentangle whether season per se or specific climatic factors are responsible for suicide seasonality^24^.

The lack of robust associations between sunshine and temperature and suicidal behaviour in our study may at least in part be explained by its unique methodological characteristics. First, our source population comprised of patients starting on antidepressant medications. We have previously shown that a consistent seasonal pattern in completed and attempted suicides does not exist in this population^25^. On the contrary, in that study a seasonal pattern was observed in suicidal behaviour in older patients with higher risk during the spring and summer and in younger patients during autumn. Interestingly, in this work we report associations between sunshine and temperature only in the older age group.

One recently formulated hypothesis by Holopainen et al. is that the over-activated brown adipose tissue may explain at least in part the associations between temperature and suicide. Brown adipose tissue (BAT) is prevalent in human adults and is responsible for cold and heat tolerance. When activated it generates heat by a process known as non-shivering thermogenesis. Rapid temperature changes activate brown adipose tissue with physiological responses that resemble the phenotype of melancholic depression such as loss of appetite, weight loss, insomnia and lack of reactivity to pleasurable activities. The long photoperiod on the other hand inhibits brown adipose tissue during the day^26^.

Temperature may be involved in the suicidal pathway in other ways, including the activation of the serotonergic neurotransmission in the brain according to animal studies, platelet [^3^H]-citalopram binding studies and 5-HT2A receptor responsiveness in humans^27,28^. 5-HT2A receptor are implicated in mood and suicidal behaviour^29^. These findings imply that temperature might modulate serotonin function and suicidal behaviour. Thus, there might be an interaction between the increase of serotonergic neurotransmission due to increased temperature and the one by the action of antidepressants in our population.

Another connection between temperature and suicidality among the elderly may be through impaired cognitive performance and decision making, as it is well established that impulsivity, aggressiveness and impaired decision making are important endophenotypes for suicide. Studies looking into the relationship between cognitive function and temperature have yielded mixed results; some found worse cognition related to cold or heat, while others did not observe any change^30^. A recent study suggested that the detrimental effects of heat stress on cognition in older people is moderated by humidity^31^.

Two previous explorative studies on sunshine and suicide, which made proper adjusting for season, have found associations of the same direction but in different lag and duration periods. Vyssoki et al., who looked at an exposure window up to 60 days before suicide, using data from Austria, reported a harmful association between daily sunshine and suicide up to 10 days before suicide, and a protective one between 14 to 60 days before suicide^21^. Papadopoulos et al. looked at an exposure window up to 10 days before suicide and found that sunshine duration the day before and the 4 days before suicide were significantly associated with an increased suicide risk in Greece^32^. Our results, even not directly comparable with those studies, do not give support to the hypothesis that sunshine has a direct triggering effect on suicide. A possible longer accumulative effect during several weeks before suicide or suicide attempt and only among older people is seen in our results. Apart from the climatic differences among these countries, a major difference in our study is that our population consists of people starting on antidepressants and that we utilized two crude exposure periods, each of 4 weeks. The only study, to the best of our knowledge, which looked into a similar population, patients starting on paroxetine, reported an association between the change of monthly sunshine and antidepressant response time^33^.

Our findings are partly in line with previous studies that find a positive association between sunshine and temperature and suicide^6^, although most of these studies do not use models that control for the effect of season^21^. This is a common problem and the main reason that other confounding seasonal factors, including social factors, such as social interaction, alcohol consumption and unemployment, and other climatic variables including aeroallergens^34,35^, that might be responsible for this association, cannot be excluded. In our study, confounders such as season, sunshine and temperature are included in our models.

Generally, our findings point out that, older individuals may be vulnerable to temperature and sunshine regarding completed suicides and suicide attempts. This is in accordance with previous studies reporting a higher risk in elderly for suicide and suicide attempt in summer when temperature is higher and sunshine longer^25^.

Serotonin transporter, (SERT) has been previously studied and was found to have a lower availability with increasing age^36^. This in combination with the fact that SERT has a lower binding capacity in the spring and summer and that treatment with an antidepressant further blocks the SERT, might contribute to a higher risk for suicide in the elderly in periods of higher temperature and more sunshine, through a redundancy of serotonin in the synaptic cleft leading to an over-activated state with anxiety and psychomotor agitation. Particularly noteworthy is that the seasonal SERT binding is accentuated in people with the short allele of the SERT^37^. This polymorphism is associated with anxiety-related traits and increased risk for depression in interaction with psychosocial adversity across the lifespan^38^.

A positive association between sunshine exposure and suicidal behavior may be also mediated by sleep disruptions and increased impulsivity that have been shown to be independent risk factors for suicidal behavior^39,40^. Unfortunately, no data on sleep patterns were available for this study.

To our knowledge, this is the first study to investigate suicidal behaviour in relation to sunshine and temperature in individuals treated with antidepressants. We used a large population-based cohort through Swedish national registers as our source population. Our analyses are comprehensive and strict compared to previous studies in the same topic, as we took into account and integrated in our models season and climatic variables as confounders thus making our results more accurate.

However, the findings of this study need to be interpreted within the context of its limitations.

An important limitation of our study is that we used information of redeeming medication as a proxy of actual antidepressant treatment, without taking into account the duration of treatment according to the amount of dispensed medication or the timely refilling of the prescriptions. We know that this proxy might not reflect the patient’s actual use of the medication as up to 60% of patients that receive a prescription do not follow the intended treatment^41^.

It cannot be excluded that other factors such as psychiatric diagnoses, which were not available for the majority of the study population who received a prescription from the primary care setting, might also have influenced our results.

In our source population, patients with combination or augmentation therapy at baseline were excluded and those receiving combination or augmentation therapy or switching to another antidepressant during follow-up, were censored at that time; thus, our results refer to patients with less severe psychopathology. Similarly, we excluded individuals receiving inpatient care during the last year before inclusion and those prescribed mood stabilizers in order to exclude patients with more severe psychopathology including bipolar disorder. Still, there is a theoretical possibility that some subjects included in our study might have an undiagnosed mixed state (agitated depression) and were treated with antidepressant monotherapy.

Another limitation is that we cannot make a distinction between non-suicidal self injury (NSSI) and suicide attempt, using register data, as there is no information on the suicidal intent in the registers.

Finally, our outcome measure of suicide attempts is a composite measure of attempts of both high and low lethality. As there are indications that lethality of the suicide attempts may moderate the association between sunshine and temperature with suicidal behaviour^42^, it would be of interest to assess these associations separately for high- and low-lethality suicide attempts. However, having a study design with multiple time windows for our exposure variables and different stratifications for sex and age, we chose not to look further into different types of suicide attempts in order not to further increase the number of performed statistical analyses. Along with an increased risk of chance findings there would also be an increased risk for false negative findings due to less statistical power. This aspect warrants further investigation in future studies.

In conclusion, even though we could not replicate previous findings of an overall association between sunshine and temperature and suicidal behaviour, our results point to a possible moderating effect of age, with higher risk of suicide attempts in the older patients.

## Methods

### Data sources

Anonymized data were obtained from the National Board of Health and Welfare and Statistics Sweden. All Swedish registers use a ten-digit national registration number (NRN), a unique personal identifier assigned to all residents in Sweden, allowing individual linkage between registers^25^.

The Swedish Prescribed Drug Register contains patient identities for all dispensed prescribed drugs in Sweden for the entire Swedish population since July 2005. The National Patient Register (NPR) has nearly complete nationwide coverage for discharge diagnoses in both somatic and psychiatric settings in Sweden based on the International Classification of Diseases (ICD). It has full coverage of all inpatient care in Sweden since 1987. Outpatient specialist visits, including psychiatric visits from both private and public caregivers, are included since 2001. Each record includes admission and discharge dates, the main discharge diagnosis and secondary diagnoses. The NPR does not cover visits in the primary care. The Cause of Death Register includes all individuals who died either in Sweden or abroad since 1952 and who were resident in Sweden at the time of death. The data are based on death certificates that provide information on date as well as underlying main and secondary causes of death using the ICD codes^25^.

The Swedish Meteorological and Hydrological Institute (SMHI) provides data on a variety of weather variables. For daily sunshine hours, we used the STRÅNG model, which calculates a number of solar radiation measurements on a grid covering northern Europe since 1999. The model grid covers the geographic area of Scandinavia with a resolution of 11 × 11 km (http://strang.smhi.se). By giving information on latitude, longitude and time period, the model gives data for daily sunshine hours in a specific location under a certain time period. For daily temperature, we retrieved data from the historic open data of SMHI, where there are available observations from actual weather stations in all 21 Swedish counties. By this way, we obtained data for average daily sunshine duration and average daily temperature during our study period in all Swedish counties.

### Study design

Nested case–control study.

### Source population

From the Swedish Prescribed Drugs Register we initially identified 1,027,666 individuals who redeemed a prescription of at least one antidepressant between July 2006 and December 2012. Patients who had redeemed a prescription of antidepressants (N06A), antipsychotics (N05A) or mood stabilizers (N03A), or who had been admitted to a psychiatric department during the year prior to inclusion were excluded, to achieve a wash-out period and focus on new treatment episodes. We excluded patients admitted to a psychiatric department during the year prior to inclusion, because information on medication treatments while in inpatient care is not available in the Swedish registers. Further, 12,532 individuals were excluded because they had a prescription of an antipsychotic or mood stabilizer at the same time as the prescription of antidepressants. An additional 202 individuals were excluded because the date of dispense was after the date of death, probably due to administrative errors. Finally, 136 individuals were excluded because of missing information in the prescription register. During the 3-month follow-up, 411 patients were censored because they moved from Sweden and 99,397 dispensed afterwards another antidepressant (N06A), mood stabilizer or antipsychotic (N03A, N05A) and were therefore censored at that date. The county of residence, which is needed for the estimation of the exposure variables was available for the years 2007–2011 and thus analyses were restricted to this period and a final population of 784,792 patients.

The source population was taken to be these 784,792 patients, with time defined as the time since the week of treatment initiation (i.e. time in cohort, ranging from 1 to 12 weeks). Week 1 was defined as the week of treatment initiation and week 12 was defined as the end of the follow-up period.

### Cases

Cases were defined as patients who attempted (ICD-10, X60–X84) or committed suicide (ICD-10, X60–X84) within 3 months from being included in the source population. The index week of cases was the week in which the suicide or suicide attempt occurred. There were 11 people who attempted suicide within the follow-up period, survived, and then subsequently committed suicide within the follow-up period. For the purposes of the suicide analysis, attempted suicide within the follow-up period was not a censoring event (i.e. these 11 people's suicides were included in the analysis and they were not censored). For the purposes of the attempted suicide analysis, a person's first suicide attempt within the follow-up period was considered to be an event and time after this event was excluded from time at risk.

### Controls and sampling of controls

Risk-set sampling was used to sample the controls. For each case, 10 controls were randomly sampled from the population at risk. As mentioned previously, time was defined as the time since the week of treatment initiation. The following procedure was repeated for each case: the week in which the case’s event (attempted suicide or committed suicide) occurred was noted (e.g. week 10). All patients who were at risk of an event in that week (e.g. were still in the cohort-at-risk in week 10 since they had not yet had a censoring event) were eligible for selection as a control. Out of this group of patients, 10 were randomly selected as controls and were recorded as being matched to the case.

### Exposures

We considered four exposures for our study: average daily sunshine and average daily temperature over the 1–4 and 5–8 week periods prior to the index week. To clarify notation: if the index week was week 10, then 1–4 weeks prior were considered to be weeks 10, 9, 8, and 7.

### Statistical analyses

Odds ratios (ORs) and 95% confidence intervals (CIs) for the risk of suicide or suicide attempt associated with sunshine hours and temperature were estimated using conditional logistic regression. All ORs were estimated for an increase of one unit in sunshine or temperature (1 h in average daily sunshine duration over a 4-week period or one degree Celsius in average daily temperature over a 4-week period respectively).

For each analysis, we ran three models:

1. The “Crude model”. This conditional logistic regression model included a single explanatory variable as exposure: either sunshine or temperature.
2. The “Base model”. This conditional logistic regression model builds upon the “crude model”. In addition to everything that the “crude model” includes, the “base model” included the following explanatory variables as confounders: previous suicide attempt, county (dummy variables), year (continuous), and season when the antidepressant treatment was initiated (three dummy variables: spring, summer, and autumn).
3. The “Climatic model”. This conditional logistic regression model builds upon the “Base model”. In addition to everything that the “base model” includes, the “climatic model” included an additional exposure of the other climatic variable (e.g. if the base model’s exposure was average daily sunshine over 1–4 weeks prior, then the climatic model would have two exposures: average daily sunshine and temperature of 1–4 weeks prior).

#### Effect modification

We were interested in the effects of possible effect modification by season, age, and sex. For each outcome (suicides and attempted suicides), exposure (average daily sunshine/temperature in the past 1–4 and 5–8 weeks), and effect modification of interest (season: three dummy variables of spring, summer, and autumn, age: two dummy variables of 25–64, and 65+, and sex: dummy variable for male), we took the base model and created two models.

- Model 1: Base model plus effect modification dummy variables
- Model 2: Model 1 plus interaction terms with the effect modification dummy variables

To make this more explicit, to test for effect modification by sex:

- Model 1: Base model + is_male
- Model 2: Base model + is_male + is_male*exposure

To test for effect modification by age:

- Model 1: Base model + 25–64 + 65 + 
- Model 2: Base model + 25–64 + 65 +  + 25–64*exposure + 65 + *exposure

To test for effect modification by season.

- Model 1: Base model + spring + summer + autumn
- Model 2: Base model + spring + summer + autumn + spring*exposure + summer*exposure + autumn*exposure

We then performed likelihood ratio tests to compare the two models and identify if the interaction terms with the exposures were statistically significant.

#### Effect estimates within strata

Using regression models with interaction terms (as specified in the “Effect modification” section), we estimated linear combinations of the estimated parameters to obtain effect estimates within strata. This is equivalent to running stratified regressions within the strata, however, it yields better estimates as all data are available for estimation of the confounders’ coefficients.

Data management of the source population was performed using SAS statistical software (version 9.1, SAS Institute, Cary, NC, USA) while all analyses were performed with R 3.3.2.

### Ethics

The study was approved by the research ethics committee in Stockholm, Sweden (Dnr 2011/1358-31/3 and 2013/1775-32) all analyses were performed in accordance with relevant guidelines and regulations.

## Data Availability

Our study includes data from health registers in Sweden which cannot be shared due to confidentiality issues. Data are available from the National Board of Health and Welfare in Sweden (registerservice@socialstyrelsen.se) and Statistics Sweden (mikrodata@scb.se).
